# ZOom Delivered Intervention Against Cognitive decline (ZODIAC) COVID-19 pandemic adaptations to the Post-Ischaemic Stroke Cardiovascular Exercise Study (PISCES): protocol for a randomised controlled trial of remotely delivered fitness training for brain health

**DOI:** 10.1186/s13063-024-08154-1

**Published:** 2024-05-18

**Authors:** Amy Brodtmann, Alex Billett, Rachael Telfer, Kim Adkins, Laura White, Laura J. E. McCambridge, Louise M. Burrell, Vincent Thijs, Sharon Kramer, Emilio Werden, Barbara R. Cardoso, Matthew Pase, Stanley Hughwa Hung, Leonid Churilov, Julie Bernhardt, Kathryn Hayward, Liam Johnson

**Affiliations:** 1 Department of Neuroscience, School of Translational Medicine, Monash University, Melbourne, VIC Australia; 2 The Florey, Melbourne, VIC Australia; 3 Department of Medicine, University of Melbourne, Melbourne, VIC Australia; 4 Austin Health, Melbourne, VIC Australia; 5 Department of Nutrition, Dietetics and Food, Monash University, Melbourne, VIC Australia; 6 Victorian Heart Institute, Monash University, Melbourne, VIC Australia; 7 Turner Institute, Monash University, Melbourne, VIC Australia; 8 Department of Physical Therapy, Faculty of Medicine, University of British Columbia, Vancouver, Canada; 9 Australian Catholic University, Melbourne, VIC Australia; 10 Epworth Rehabilitation, Melbourne, VIC Australia

**Keywords:** Ischaemic stroke, Dementia, Cognition, Exercise training, Physical activity, Telerehabilitation, Brain volume

## Abstract

**Background:**

Stroke increases subsequent dementia risk yet there are no specific post-stroke therapies to protect cognition. Cardiorespiratory exercise is recommended for secondary prevention of stroke and may be neuroprotective. The Post Ischaemic Stroke Cardiovascular Exercise Study (PISCES) aims to reduce post-stroke secondary neurodegeneration and cognitive decline. During the pandemic, we pivoted to a ZOom Delivered Intervention Against Cognitive decline (ZODIAC) protocol, reducing pandemic-amplified barriers to exercise.

**Methods:**

We present pandemic adaptions for a multicentre phase IIb assessor-blinded randomised controlled trial of ischaemic stroke survivors testing the efficacy and feasibility of an 8-week home-based exercise intervention delivered at 2 months post-stroke. We compare cardiorespiratory exercise (intervention arm) versus balance and stretching (active control arm). Participants are assessed with magnetic resonance imaging (MRI), fitness, blood, microbiome, and neuropsychological tests at three study visits: before and after the exercise intervention and at 12 months. Modifications to the original protocol include pre-exercise safety home visits, commercial delivery of exercise equipment to facilitate assessor blinding, and reconsideration of statistical plan to allow pooling of the studies. We have reduced in-person study visits from 27 to 3. Primary outcome remains between-group (intervention versus control) difference in brain volume change; secondary outcome is between-group difference in global cognitive ability to allow remote administration of a validated cognitive scale.

**Discussion:**

Remotely delivered exercise interventions reduce participant burden and may reduce barriers to recruitment. A decrease in the number of in-person study visits can be supported by greater information capture via self-reported questionnaires and phone surveys.

**Trial registration:**

Prospectively ACTRN12616000942459. Registered on July 2016.

**Supplementary Information:**

The online version contains supplementary material available at 10.1186/s13063-024-08154-1.

## Introduction

### Background and rationale {6a}

Dementia is a global health challenge affecting approximately 50 million people [1]. Guidelines for dementia prevention align closely with those promoting cardiovascular health [2]. Stroke is a major risk factor for dementia, with incidence of dementia almost 50 times higher in the first year after stroke compared to individuals who have not had a stroke [3]. In our Cognition and Neocortical Volume after Stroke (CANVAS) study [4], we demonstrated that stroke is associated with accelerated rates of structural brain ageing, including atrophy and white matter hyperintensity burden [5, 6]. Brain atrophy is the hallmark of neurodegeneration and correlates with cognitive decline [7].

Cardiorespiratory exercise is recommended after stroke for secondary prevention of cardiovascular disease via risk factor modification [8, 9]. Cardiorespiratory exercise can improve cardiac function, hypertension, insulin sensitivity and fitness following stroke [10] and may also improve post-stroke cognition [11]. Despite these known benefits, people with stroke are commonly inactive, unfit [12] and at significant risk of further cardiovascular disease and dementia [13]. People with stroke have limited opportunities and support for essential rehabilitation and secondary prevention beyond initial inpatient therapy [13]. Cardiorespiratory exercise following stroke is seldom prescribed clinically. A lack of community-based exercise programs, especially for those residing in rural/remote areas, and access barriers to attend these programs further limit uptake of cardiorespiratory exercise training post-stroke [14]. These barriers were accentuated by lock-down restrictions during the COVID-19 pandemic, which were severe in Melbourne, Australia, with the longest lockdown in the world [15].

Telerehabilitation, the delivery of rehabilitation services via electronic networks, offered a solution to overcome these barriers. In stroke, it is as effective and feasible as in-person therapy [16], and patients report that telerehabilitation is also enjoyable [17]. Additionally, remotely delivered cognitive assessments using videoconferencing platforms have been validated in neuropsychology and used in similar studies [18]. There is growing evidence that remotely delivered exercise may encourage participation for those who face long distances to travel and those with caregiver and family responsibilities that limit their capacity to engage in centre-based rehabilitation [19]. These environmental and social barriers are common in older adults, especially remote and rural Australians. There is also encouraging evidence that remotely delivered cardiac rehabilitation and exercise interventions produce comparable health-related quality of life outcomes [20], associated with comparable adherence [21] to face-to-face interventions.

The Post-Ischaemic Stroke Cardiovascular Exercise Study (PISCES) [22] was designed as a randomised controlled trial of fitness training for brain health. Time constraints and accessibility were identified as common barriers to recruitment—factors that were exacerbated by the pandemic. We present PISCES protocol modifications to offer a remotely delivered intervention: a ZOom Delivered Intervention Against Cognitive decline (PISCES-ZODIAC). The reduction of in-person study visits from 27 to 3 aimed to reduce participant burden and pandemic-amplified barriers to participation. This reduced study visit schedule allowed us, the investigators, to “value-add” to our in-person visits and include other self-administered scales.

### Objectives {7}

To test the hypothesis that stroke survivors receiving an at-home cardiorespiratory training intervention will experience a lower rate of decline in hippocampal and total brain volume at 4 months post-stroke (i.e. preserve brain volume) compared to stroke survivors receiving balance and stretching training (non-aerobic, active control).

### Trial design {8}

Post Ischaemic Stroke Cardiovascular Exercise Study—Zoom Delivered Intervention Against Cognitive Decline (PISCES-ZODIAC) is a multicentre, phase IIb assessor-blinded, randomised (1:1 allocation) controlled trial testing the preliminary efficacy and feasibility of an 8-week home-based exercise intervention modelled on cardiac rehabilitation, delivered at 2 months after stroke. Participants are assessed before (at 2 months post-stroke: t_1_) and after (at 4 months post-stroke: t_2_) the exercise intervention and again at 12 months post-stroke (t_3_). We will report the trial according to the Standard Protocol Items: Recommendations for Interventional Trials (SPIRIT) guidelines (Table 1) [23] and the Template for Intervention Description and Replication (TIDieR) [24]. It is registered with the Australian and New Zealand Clinical Trials Registry (ANZCTN12616000942459).

**Table 1:** Standard Protocol Items: Recommendations for Interventional Trials (SPIRIT) diagram of the study procedures for the PISCES-ZODIAC trial

	Study period			
	Enrolment	Pre- intervention	Post-intervention	Follow-up
Time point	t_0_	t_1_	t_2_	t_3_
Enrolment				
Eligibility screen	X			
Informed consent	X			
Randomisation		X		
Home safety visit		X		
Delivery of equipment		X		
Intervention				
Cardiorespiratory (Active Intervention)				
Balance and Stretching (Control Intervention)				
Assessment				
Brain MRI		X	X	X
Cognition*		X	X	X
Clinical scales*		X	X	X
Demographics		X	X	X
Mood and quality of life*		X	X	X
Physical fitness*		X	X	X
Sleep*		X	X	X
Cardiovascular risk*		X	X	X
APOE genotyping		X		
Blood biomarkers*		X	X	X
Dietary intake		X	X	X
Gut microbiome		X	X	X

*MRI* magnetic resonance imaging, *APOE* apolipoprotein E gene

^*^Refer to Supplementary Table 1 for detailed assessments

## Methods: participants, interventions and outcomes

### Study setting {9}

Participants are recruited from four metropolitan university teaching healthcare networks in Melbourne, Victoria, Australia: Austin Health, Eastern Health, Epworth Health, and Western Health. Central ethics approval has been granted by the Austin Health Human Research Ethics Committee (lead site, HREC/16/Austin/45), with site-specific governance approval from others (most recent approval 07 September 2023, Protocol_v14). Austin, Eastern and Western Health sites are public hospital networks, whilst Epworth Health is a private hospital network with sites throughout the state of Victoria.

### Eligibility criteria {10}

The patient population remains the same in the current protocol: adult ischaemic stroke survivors without severe prior functional impairment (i.e. premorbid modified Rankin Scale ≥ 3) or any reported cognitive impairment. The inclusion and exclusion criteria are summarised in Table 2. We placed a 1-h travel radius around Melbourne Central Business District for our participant residence catchment (~ 100 km) for logistical reasons (courier truck delivery costs for exercise equipment; attendance at three in-person study visits).

**Table 2:** PISCES-ZODIAC inclusion and exclusion criteria

Inclusion criteria	Exclusion criteria
• Ischaemic stroke	• Significant medical co-morbidities precluding participation in an exercise intervention (e.g. severe cardiac disease), or making survival at 12 months unlikely
• Premorbid mRS of ≤ 3	• Contraindications for MRI (e.g. implanted metal, severe claustrophobia)
• Able to give consent	• Pre-existing cognitive decline or psychiatric disorder
Aged ≥ 18	
• Able to nominate an emergency contact person	

*PISCES-ZODIAC* Post-Ischaemic Stroke Cardiovascular Exercise Study—Zoom Delivered Intervention Against Cognitive Decline, *mRS* modified Rankin scale, *MRI* magnetic resonance imaging

### Who will take informed consent? {26a}

Information flyers are handed to potential participants by clinical staff where appropriate. Study staff perform screening remotely via accessing hospital discharge summaries or direct notification from clinical staff (e.g. inpatient stroke clinicians on acute or rehab ward). Participants are contacted for their interest following discharge. Initial consent is obtained via video-call, phone call or in-person visit. Further information is then obtained regarding MRI eligibility (e.g. medical notes for prior procedures, etc.) after initial consent. Formal written consent is provided at first study visit. Randomisation occurs after the first MRI and their study schedule is arranged.

### Additional consent provisions for collection and use of participant data and biological specimens {26b}

Consent is given for all study procedures. Optional consent is available for sharing MRI images with other studies and for DNA storage for sharing with international consortia.

## Interventions

### Explanation for the choice of comparators {6b}

The training intervention is modelled on cardiac rehabilitation, which includes a professionally supervised, 6–10-week exercise training program and has been shown to improve multiple cognitive domains, including memory, attention and global cognition in people with cardiovascular disease. We included an active control arm for two reasons: to maintain blinding for the participant (as a placebo arm could mean no intervention which would unblind the participant) and to encourage recruitment and adherence, as randomisation to no intervention may not be appealing. We emphasise that the key prescription parameters of the intervention (i.e. cardiorespiratory exercise intensity, duration and frequency) remains unchanged except for the mode of delivery. The intervention and active control groups have been previously described [22].

### Intervention description {11a}

#### Home safety assessment

Once intervention arm is allocated, the home safety visit is arranged to allow delivery of and familiarisation with the exercise equipment. A trained exercise professional (TEP) performs a home visit and safety assessment for every participant, prior to commencing either arm of the exercise program. A comprehensive home safety assessment checklist was developed using clinical home visit standards and protocols used by Austin Health clinicians (see acknowledgements) and used to guide each safety assessment. The participant’s level of function determines if they can complete the exercise protocol safely on their own or if they will require a ‘study partner’ (e.g. family member, neighbour, friend) to be present. The TEP and the participant also establish a safe exercise space and an appropriate location for videoconferencing. All TEPs strictly abide by current Australian Department of Health and Human Services requirements and local hospital governance guidelines, including the use of Personal Protective Equipment as required.

#### Appropriate equipment delivery

All equipment required to complete the intervention is delivered either by a professional courier company (larger items, e.g. bike) or the TEP (smaller items, e.g. mats, weights) (see Table 3 for full equipment list). The TEP familiarises the participant with the training protocol, exercise equipment and the technological aspects (i.e. iPad, data plan and *Zoom*). Equipment for the aerobic exercise arm (Steelflex PB10 Upright Bike or Steelflex CBSG Artiso Commercial Upright Bike) is stored offsite in a secure storage facility nearby our professional couriers. Once treatment arm is allocated, couriers are notified to collect and deliver large equipment (e.g. exercise bike) to the participant. This is to prevent unblinding of study staff members should they witness the arrival of a courier vehicle to our offices.

**Table 3:** Equipment provided to participants for the exercise intervention

Cardiorespiratory intervention	• Upright exercise bike (Steelflex PB10 Upright Bike or Steelflex CBSG Artiso Commercial Upright Bike)
	• Rubber dumbbell sets
	• Latex resistance bands
Balance and stretching active control	• 5-mm yoga mat
	• Safety marker cones
Both groups	• Safe to exercise measures:
	o SpO_2_ monitor (Nonin Onyx Vantage 9590 pulse oximeter)
	o Blood pressure monitor (Omron blood monitor Hem7121)
	o Fitness watch and heart rate monitor (Polar H9 heart rate sensor and Polar Unite fitness watch)
	• iPad with 4 g capability
	• Aerobic stepper

#### Safe-to-exercise measures and monitoring

Safe-to-exercise measures including blood pressure (BP), resting heart rate and oxygen saturation (SpO_2_) are taken before and after each exercise session. The participant is trained by the TEP during the home safety visit to complete these measures on their own or with assistance (study partner) under the observation (via *Zoom*) of the TEP.

For safety purposes and to measure intervention fidelity, during each exercise session, the participant will self-monitor and report their heart rate (HR) and the Borg Rating of Perceived Exertion [25] back to the TEP in real time. If a participant experiences a dose-limiting event (e.g. a participant does not pass safe-to-exercise measures or experiences an adverse event during a session), rules are in place to manage and adapt the exercise training within safety limits to encourage continued participation.

#### Telerehabilitation delivery

A TEP delivers and monitors the participant’s exercise sessions via *Zoom* three times a week for 8 weeks. Each training session consists of up to 60 min of exercise, beginning and ending with a 5-min warm up and cool down, unchanged from the previous protocol.

#### Cardiorespiratory exercise intervention and the balance and stretching groups

The intervention and active control groups have been previously described [26]. We emphasise that the key prescription parameters of the intervention (i.e. cardiorespiratory exercise intensity, duration and frequency) remain unchanged except for the mode of delivery. Participants randomised to the *intervention group* complete up to 32 min of cardiorespiratory exercise on a stationary bike three times per week (not treadmill alternative in view of falls risk at home). This includes two moderate-to-high intensity interval sessions and one continuous steady-state session per week. Participants also complete twice weekly 15-min bouts of moderate intensity strength training during the cardiorespiratory interval training sessions. Strength training progression is based on participant tolerance, with the aim for participants to maintain an RPE of 12 to 15 (i.e. light to hard).

Participants in the *active control group* complete 30 min of light, static stretching and 20 min of static and dynamic balance-based exercises three times per week. The protocol for this group was designed to match the attention and dose (i.e. session duration and frequency) the participants in the intervention group receive, however limits exercise intensity that could potentially lead to improvements in cardiorespiratory fitness. Supplementary Table 2 outlines an example of a weekly schedule and exercises for both intervention and active control groups.

### Criteria for discontinuing or modifying allocated interventions {11b}

Participants are instructed that they can discontinue at any time. Interventions are modified according to the participant’s post-stroke disability and pre-existing conditions guided by TEP. Our exercise professionals all have tertiary qualifications in administering exercise in patient populations.

### Strategies to improve adherence to interventions {11c}

Interventions are administered one-on-one via Zoom. Participants are observed throughout the interventions and provide verbal feedback to the TEP. Notes are taken continuously on participant experience. Exercise staff members are trained in motivating patient populations.

### Relevant concomitant care permitted or prohibited during the trial {11d}

All usual medical care is continued during the trial. There are no medications or procedures that are prohibited.

### Provisions for post-trial care {30}

Australia has universal health care, so participants have access to ongoing Medicare care. Trial participants are covered by university indemnity insurance for trial associated serious adverse events.

### Outcomes {12}

*Primary outcome* is unchanged: between-group (intervention versus control) difference in hippocampal and total brain volume change between 2 and 4 months post-stroke (i.e. pre- and post-intervention).

#### Secondary outcome (changed)

Differing from the original protocol, the main cognitive outcome measure (secondary outcome) is global cognitive ability as measured by the Alzheimer’s Disease Assessment Scale-cognition sub-scale (ADAS-Cog) [27] at 12 months/study visit 3.

This change was made to capture global cognition with a validated scale that can also be delivered remotely. The ADAS-Cog measures several cognitive domains: memory, language, praxis and orientation. It is administered at 2 and 12 months post-stroke, in addition to neuropsychological testing (see Supplementary Table 1), which is delivered at each study visit. Our cognitive outcome previously was executive function as measured by Trail Making-B test performed in the neuropsychological testing battery at each study visit. This executive score is now an exploratory outcome.

### Exploratory outcomes

The relationship between cardiorespiratory fitness, cognitive domains, physical activity, 24-h ambulatory BP, recurrent stroke, pre-specified growth factors and *APOE* ε4 allele status continue to be investigated with the following additions:

- Intervention fidelity for each session for cardiorespiratory participants is achieved (yes/no) if exercise intensity (mean % of heart rate reserve) and time (minutes ± 5%) meet the prescription. Attendance is met if participants attend ≥ 20 sessions of 24 scheduled.
- Measures related to mood, sleep, cardiorespiratory fitness, cardiovascular health and daily physical activity are described in the original protocol [26] and listed in Supplementary Table 1. Additional to these measures are the following:
  o Inflammatory markers: venous blood is drawn to analyse inflammatory biomarkers (IL-6, IL-1β, TNF-α, IL-8, IL-10 and IL-1ra) and neurodegenerative markers (neurofilament light chain, NfL)
  o Dietary intake: participants complete a 3-day food diary using a smartphone application (Research Food Diary, Xyris Software, Australia xyris.com.au). This is shared with the study nutritionist (BRC). Nutrient analysis is undertaken using an Australian food composition database, the FoodWorks Professional (v10) software.
  o Gut microbiome: participants are provided with a take-home stool specimen collection kit, including storage tubes with a DNA preserving agent, and instructions. The sample must be from the first bowel movement of the day and not collected on a day that participants are also completing the food diary. The stool sample is stored in a thermo insulated bag in the freezer until it can be returned to the research team within 24 h. Samples are stored in − 80 °C freezers until microbial sequencing takes place to examine the gut microbiome composition.

### Specific pandemic-driven changes to the original protocol

#### Cardiorespiratory fitness—removal of VO_2peak_.

PISCES fitness testing via the graded exercise test conducted on a total body recumbent stepper (NuStep, T5XR, NuStep, Inc., Ann Arbor, MI) estimated peak volume of oxygen consumption (VO_2peak_) via a gas analysis system. To minimise the risk of aerosol transmission whilst COVID-19 lockdown restrictions were in place, VO_2peak_ was predicted via an equation utilising heart rate [28]. COVID-19 hospital infection control protocols were followed until mid-2022 when government COVID-19 restrictions were fully eased. Upon easing of restrictions, we were permitted to return to a breath-by-breath pneumotach gas analysis system (Jaeger® Oxycon Mobile) to measure VO_2peak_ at one site only and remained prohibited from its use at our other sites. In view of this, we have removed the VO_2peak_ as an outcome measure and replaced it with a prediction equation developed in older adults to estimate VO_2peak_ using a total body recumbent stepper [29]. We note that this equation will require optimisation for our participants. It was developed on community volunteers, potentially biasing the results toward fitter, more motivated participants. Submaximal effort due to health anxiety and inability to exercise arising from musculoskeletal issues will impact fitness assessments. We note that the energy cost of steady-state activity is greater in stroke survivors [30], which could also potentially affect our estimation.

#### Physical activity measure changed to Actiwatch

The technology used to measure daily physical activity has been changed to the Actiwatch Spectrum Plus (Koninklijke Philips, Amsterdam, Netherlands). This change was because they are relatively light weight, travel well via postage and could be given with a return padded envelope to be mailed back by the participant. Additionally, the Physical Activity Scale for the Elderly (PASE) questionnaire [31] is utilised weekly throughout the 8-week intervention to monitor participants’ physical activity outside their scheduled sessions as well as phone-delivered twice monthly between study visits 2 and 3. We included this to maintain participant interest in the study, given that study visits and in-person access to our TEPs had reduced with our new study design. It also allowed us to monitor self-report of their activity between the 4- and 12-month visits.

### Participant timeline {13}

Study visits and procedures are outlined in Table 1. Study visit 1 occurs at 2 months post-stroke, prior to the exercise intervention which is required for randomisation to intervention arm as total brain volume is used in the stratification schedule (see below). Each study visit includes MRI brain, blood and stool samples, fitness and cognitive testing. Study visits occur pre- and post-intervention and at 12 months post-stroke. Participants are contacted via phone call for administration of the Physical Activity Scale for the Elderly (PASE [31]) to maintain study contact and increase retention.

Outcome measures for each time point are listed in Table 1 and Supplementary Table 1. To accommodate a range of participant needs, and to adapt to COVID-19 restrictions and risk of infection, participants are given the option to complete the questionnaires and neuropsychological test battery either face-to-face or remotely, via the *Zoom* (www.zoom.us) videoconferencing platform. Participants make this decision prior to their baseline assessments, and the delivery mode remains consistent across their subsequent time points. Participants that choose to complete the assessments remotely receive a hard copy remote cognitive assessment package in the post, including detailed instructions. Some of the assessments are enclosed in a separate envelope which is opened on request whilst in the testing session. Supplementary Table 1 details summarises mode of delivery of study procedures.

### Sample size {14}

The database for the 34 PISCES participants was locked at their final study visit (10 December 2020). Recruitment for PISCES-ZODIAC commenced in November 2020. We aim to pool data in our analyses as well reporting individual datasets.

Sample size estimation and statistical analysis plan was reconsidered from the original PISCES protocol [26]. Originally, a total sample size of 100 participants (50 per group) was calculated to yield 80% power to detect a difference in hippocampal volume between groups corresponding to a medium-to-large effect size (*d* = 0.6), assuming the standard settings of two-tailed significance and alpha = 0.05. In CANVAS [5], we found hippocampal volume loss of − 3% over 4 months after stroke, in contrast to the − 0.4% change in the stroke-free control group. This gave us a total of 45 patients per group. We increased the sample size by 10% originally to account for attrition over the 12-month period and the possibility of non-evaluable scans. We estimated a potential increase in attrition rates to 20% as there were rolling lockdowns including curfews and limitations of travel (5-km radius at some stags) and potential COVID-19 infection affecting ability to participate in the intervention or attend assessments. We were reassured by multiple studies where remote exercise interventions were not inferior to in-person on measures of adherence, safety, feasibility and health quality of life [20]. Hence, our PISCES-ZODIAC sample size increased to 110 (45/group + 20) participants, still requiring a total of 90 completed participants overall.

### Recruitment {15}

Team logistics meetings occur weekly. All team members attend weekly laboratory meetings in addition to logistics meetings. Patient lists from each hospital are screened at least twice weekly. A log of all participants screened is kept which includes those potentially eligible. Reasons for exclusion are annotated.

### Assignment of interventions: allocation

#### Sequence generation {16a}

Randomisation and blinding protocols have been described in Johnson, Werden [26], and remain the same in the current protocol, except for added precautions for delivery of exercise equipment. In brief, after study visit 1, randomisation occurs following MRI. Randomisation is stratified by baseline modified Rankin scale (mRS, 0–1 versus 2–3) [32] and baseline total brain volume (less than 1 million mm^3^ versus greater than or equal to 1 million mm^3^, grouped into high or low total brain volume based on the median 2-month total brain volume derived from the CANVAS dataset [4]). This was to endeavour to have balanced functional status for participants and to ensure that participants with larger head sizes (and therefore larger brains) were not allocated unequally to one intervention arm by chance. Permuted blocks of undisclosed size are used within individual strata to ensure sequential balance and to maintain blinding. Block sizes will be disclosed in the final manuscript after the study database lock.

#### Concealment mechanism {16b}

Randomisation schedule is managed by a staff member not involved in the PISCES-ZODIAC study. Participants are assigned a deidentified participant ID (PID) on study entry and this is used for all database entry.

### Implementation {16c}

Stroke inpatient and discharge lists are screened for potential participants by Research Assistant staff, who enrol participants. Generating allocation sequence and assigning participants to interventions is part of the randomisation process (see above), conducted by staff not involved in the study. TEPs deliver the intervention.

### Assignment of interventions: blinding

#### Who will be blinded {17a}

PID is used for all data input and analyses.

Following study visit 1 MRI scan, the MPRAGE images used to generate total brain volume are shared with our image analyst using this PID. TBV (number) is shared with the independent staff member for the randomisation schedule and the TEP is instructed regarding intervention arm. There is no communication with assessors or image analysts regarding intervention allocation.

TEPs are not blinded to intervention arm but are instructed not to discuss any intervention details with the study assessors completing assessments. Participants are instructed to only discuss their intervention with the TEPs. A TEP not involved in the intervention is employed to perform the fitness assessment at study visits. We have employed permanent TEPs for intervention delivery and utilise casually employed TEPs for study visits. Participants are informed that they will be allocated into one of two intervention arms. Details are not given about either arm to maintain participant blinding by comparison with the alternative arm, but participants are of course aware of the exercises they are being given.

#### Procedure for unblinding if needed {17b}

The design is open label with only outcome assessors being blinded so unblinding will not occur. Unblinding will occur at study close. Unblinding can also occur at the request of the medical monitor, another member of the Data Safety Monitoring Committee or the participant’s treating doctors.

### Data collection and management

#### Plans for assessment and collection of outcomes {18a}

Study instruments are listed in Supplementary Table 1 and in the schedule in Table 1.

#### Plans to promote participant retention and complete follow-up {18b}

Thrice weekly sessions with our TEPs and 2-monthly phone calls for administration of the PASE are designed to promote patient retention. All efforts are made to arrange transport for participants (e.g. taxi vouchers, Uber bookings) or complete assessments remotely. Bi-annual updates regarding the project are shared with participants as well as any recent media coverage.

Participants who withdraw prior to any study procedures (i.e. initial consent for study but then withdraw prior to MRI, etc.) will not be included in the statistical analyses but will remain in the study database. Participants who have commenced study procedures but then withdraw will be included in our intention-to-treat analyses. We have set a dose of 80% of the intervention as our threshold. Participants who have completed study visits 1 and 2 but do not wish to return for study visit 3 (12 months post-stroke) are offered assessment remotely or via telephone for the administration of the ADAS-Cog (secondary outcome).

#### Data management {19}

All study staff are GCP compliant. Duties for all staff members are entered into a delegation log. New study assessors are onboarded via addition to the delegation log and shadowing experienced assessors for 4 weeks. Training on cognitive assessments was overseen by an experienced clinical neuropsychologist (EW, CR, see “Acknowledgements”). Data is entered into a REDCap database for all study visits. A separate REDCap database is used for data entry of the exercise intervention to prevent inadvertent unblinding. TEPs have access to both databases for data entry.

Quality assurance and source verification occurs at regular intervals (4–6 weekly) and double data entry is performed retrospectively by a team member not involved in the study (LCa, see ‘Acknowledgements’).

#### Confidentiality {27}

Personal information is kept in a secured, locked database. Paper copies of tests/score sheets including identifiable information is transported between study sites by study staff and filed in locked filing cabinets in office space. Participant information from the Western Hospital is kept at the study site until study close (local HREC stipulation). Images are transferred electronically using PID.

All data will be kept securely in office space or archived for 15 years after study close.

#### Plans for collection, laboratory evaluation and storage of biological specimens for genetic or molecular analysis in this trial/future use {33}

Blood and stool samples are taken at each study visit—see Table 2 and Supplementary Table 1, and outcome measurements detailed above. Blood is taken for *APOE* testing at study visit 1. Participants can consent to the study but refuse either or both blood and stool sample collection.

Storage of DNA for future analyses is offered as optional and the participant consents separately for this study component. The Participant Information and Consent Form (PICF) states:

“The blood sample will be stored in a locked freezer in the study laboratory only accessible by the study researchers and later analysed for genetic factors and blood markers that may be important in the development of dementia or heart disease. The blood test is an optional part of this study and requires separate consent. You can still participate in this study even if you choose not to consent to a blood sample being taken.”

And also:

“It is anticipated that future research in dementia will generate further new genetic and blood markers for measurement. At the current point in time, research pathways into such markers and their measurement techniques are not known. If and when the Investigators of this study become involved in such research, an application for approval to use your blood sample will need to be made to the Reviewing Human Research Ethics Committee. The purpose of this application will be to ensuring that no adverse events (i.e. no impact on your health, well-being, quality of life, or socio-economic status) are incurred. In the case that this approval is given, your stored blood sample will enable us to study new and future markers and will be kept until it is all used up. In the case that your blood sample is used, you will remain de-identified. Your blood samples will be used for research purposes only. If at any point you wish to have your samples withdrawn from potential usage then this can be discussed with the Principal Investigator.”

### Statistical methods

#### Statistical methods for primary and secondary outcomes {20a}

The primary outcome will be analysed on an intention-to-treat basis. To account for variance between PISCES and PISCES-ZODIAC, we will use mixed-effects regression models with trial and trial-by-treatment interaction terms incorporated as random effects in all models. The difference in hippocampal volume from baseline to 4 months will be compared using a linear mixed-effect regression model with group as a factor (intervention versus control) and baseline total brain volume and functional status (mRS 0–1 versus mRS 2–3) as co-variates. Effect estimates for the pooled dataset as well as for individual components (PISCES + PISCES-ZODIAC) will be reported with 95% confidence intervals.

Separate mixed-effects regression models, with groups as a factor and baseline total brain volume and functional status as co-variates, will be used to analyse the difference in hippocampal volume, total brain volume, and executive function from baseline to 1 year. A repeated measures random effect regression model will be used to determine the relationship between hippocampal and total brain volume and executive function.

#### Interim analyses {21b}

A blinded interim intervention fidelity for the prescribed exercise intensity and time was performed for PISCES and presented at the Stroke Society of Australasia Annual Meeting in 2019 [33]. A blinded interim safety analysis was conducted using the brain and hippocampal volumes of the first 20 participants in PISCES and compared to a natural history study of post-stroke brain volume [34] to ensure no harms were being done by the exercise intervention. There are no planned interim analyses for ZODIAC.

#### Methods for additional analyses (e.g. subgroup analyses) {20b}

The tertiary and exploratory outcomes will be analysed according to standard statistical principles for comparison of change between groups and associations between variables. As one of these exploratory analyses, we anticipate investigating the magnitude of the difference in various outcomes of interest (e.g. brain volume) between any PISCES/PISCES-ZODIAC exercise intervention (active or control) and historical controls (no intervention, observational cohort) from the CANVAS study.

#### Methods in analysis to handle protocol non-adherence and any statistical methods to handle missing data {20c}

Protocol deviations are logged contemporaneously with each study visit or intervention session as applicable. We expected 20% attrition. We will report final numbers in our primary outcome paper, but briefly, the current attrition rate is in line with expectations. All cases are classified as missing completely at random (MCAR) or missing at random (MAR). We will determine whether demographic and stroke characteristics are different to those of patients that withdrew from the study or were lost to follow-up.

#### Plans to give access to the full protocol, participant level-data and statistical code {31c}

The full protocol is freely available at the ANZ Clinical Trials website. The statistical analysis plan will be completely by the end of 2024 and uploaded to this website as well as included in any manuscript submissions. This will be updated with statistical code as developed. Participant level data will be available for sharing 2 years after publication of the primary outcomes.

### Oversight and monitoring

#### Composition of the coordinating centre and trial steering committee {5d}

All study procedures follow Good Clinical Practice (GCP) guidelines, and all investigators with participant-facing roles have valid GCP certification and are placed on our delegation log. An Operations Committee and a Steering Committee consisting of members of the investigation team and other trial staff convene at regular intervals. Monthly investigator meetings are held to update study staff.

#### Composition of the data monitoring committee, its role and reporting structure {21a}

A Data Safety Committee monitors trial performance, protocol violations, data quality and unblinded serious adverse events (SAEs) for trial safety bi-annually. The Data Safety Committee comprises our medical monitors and biostatistician (LC) who liaise with the principal investigator (AB) and senior investigators (KA, JB, LJ) for any questions. All staff members continue to be blinded to participant arm.

#### Adverse event reporting and harms {22}

SAEs are reported to our Data Safety Medical Monitor within 24 h of notification. Staff members complete the Adverse Event form and this is emailed to our Medical Monitors for adjudication.

#### Frequency and plans for auditing trial conduct {23}

Australian trials involving hospital participants are governed by the National Clinical Trials Governance Framework (NCTGF). Officers randomly perform assessments on any hospital or healthcare network HREC approved study. These are called Short Notice Accreditation Assessments (SNAP). PISCES-ZODIAC has not been selected for a SNAP. We regularly review the Short Notice Self-Assessment Checklist Tool. The project management group/steering committee meet monthly in the Investigator Meeting.

#### Plans for communicating important protocol amendments to relevant parties (e.g. trial participants, ethical committees) {25}

Changes to study staff are communicated to the HREC via a change to study staff form. Changes to the protocol require submission to central and local HRECs. Participants are re-consented to the current protocol if changes occur over their study participation. Investigators are updated in the monthly meetings regarding changes to eligibility criteria, paper submissions and protocol.

#### Dissemination plans {31a}

PISCES protocol was published in 2018 [22] and blinded interim fidelity data were presented in 2019 [33] (see Interim Analysis). An analysis of all participants screened and reasons for eligibility is in progress.

We plan for database lock and primary outcome analysis in the first half of 2025, with presentation at international stroke conference that year (e.g. European Stroke Organisation Conference, World Stroke Organisation) with publication shortly after. Media release will be actioned once any embargos are lifted. Results will be communicated to all participants via an emailed newsletter.

Results will also be disseminated widely on a national basis with presentation at Australasian meetings, e.g. Australian and New Zealand Stroke Organisation Conference, Australian Dementia Research Forum. We plan to convene national roundtables with stakeholders on exercise recommendations after stroke and with international peak bodies (e.g. International Stroke Recovery and Rehabilitation Alliance).

## Discussion

Exercise interventions offer a unique brain health opportunity, with potential benefits to cardiovascular fitness, cognition, mood and stroke prophylaxis. However, exercise prescriptions for brain health have not been available for stroke survivors due to insufficient evidence for recommendations on dose, timing, type, intensity and timing. The COVID-19 pandemic severely impacted global stroke care and was devastating to trial recruitment in most affected regions. Our pandemic-drive adaptions to create the PISCES-ZODIAC protocol may provide enduring solutions to barriers to recruitment for exercise interventions after stroke as well as suggestions for study assessment tools. We will provide valuable data on the effects of exercise interventions on metrics of brain health following ischaemic stroke. We aim to identify exercise interventions that prevent post-stroke and cerebrovascular cognitive decline and dementia.

## Trial status

In May 2020, 34 participants had completed the original PISCES protocol at time of severe COVID-19 lockdowns in Melbourne, Australia. The database for these participants was locked at the final timepoint (10 December 2020), allowing the data to be accessed for statistical analyses once PISCES-ZODIAC is completed.

Approval for the new PISCES-ZODIAC protocol was granted 27 October 2020, conditional on Department of Health and local hospital guidelines for the resumption of medical trials during the COVID pandemic. Recruitment for PISCES-ZODIAC commenced January 2021.

Current version approval including new study staff, the addition of a new scanning site and recruitment site was received on 07 September 2023: Protocol_v14. Recruitment for new participants continued until end of December 2023. That is, participants were eligible if their ischaemic stroke occurred up until midnight on the 31 December 2023. Final interventions will be delivered March–May 2024. Primary outcome study visits (brain volume change) will be completed April–May 2024, with secondary outcome visits/final study visits (ADAS-Cog, cognitive outcome) until December 2024.

The original protocol for PISCES was published in 2018 [22]. This amended protocol for PISCES-ZODIAC was submitted to *Trials* in December 2023, prior to study recruitment completion. The late submission of this protocol reflects the dynamic nature of our trial, pandemic-induced staff changes meaning multiple HREC updates (e.g. for new staff additions) and our desire for the protocol to be finalised prior to submission.

### Supplementary Information

Supplementary Material 1.

Supplementary Material 2.

Supplementary Material 3.

## Data Availability

All investigators will have access to the final trial dataset. As this is an RCT, no data will be publicly available until primary and secondary outcomes are published. Deidentified data will be available for sharing with researchers for registered systematic reviews and meta-analyses. Participants have an option to have their data available for sharing with researchers and only participants who have checked this box will have their data uploaded to shareable database.
