# Supplementary material for: ZOom Delivered Intervention Against Cognitive decline (ZODIAC) COVID-19 pandemic adaptations to the Post-Ischaemic Stroke Cardiovascular Exercise Study (PISCES): protocol for a randomised controlled trial of remotely delivered fitness training for brain health
Source: Trials. 2024 May 18;25:329. doi: 10.1186/s13063-024-08154-1 (PMC11102145; doi:10.1186/s13063-024-08154-1)
Supplement: Supplementary file 1 — Supplementary Material 1. [file 13063_2024_8154_MOESM1_ESM.docx]

**Supplementary Table 1.** PISCES-ZODIAC measures, methods of data collection, and availability of remote assessment option

| **Outcome Measures** | **Method of Data Collection** | **Remote assessment optionally available** |
| --- | --- | --- |
| Brain volume / hippocampal volume | 3T MRI Scanner | ✗ |
| Cognition*  *Global cognitive ability:*  *Processing speed*  *Attention*  *Memory*  *Language*  *Visuospatial function*  *Executive function* | Alzheimer’s Disease Assessment Scale-Cognitive (ADAS-Cog) Subscale  Montreal Cognitive Assessment (MoCA)  Digit Symbol Coding Task – (WAIS-IV)  Detection Task – Computerised Cogstate Battery  Digit Span Task  Trail Making Test (Part A)  Identification Task – Computerised Cogstate Battery  One-Back Task – Computerised Cogstate Battery  Hopkins Verbal Learning Task (HVLT) – Revised  Rey Complex Figure Task – Delayed Recall  Controlled Oral Word Association Test  Verbal Fluency Task (Animals)  Boston Naming Task  Rey Complex Figure Task – Copy  Cancellation Task (WAIS-IV)  Trail Making Test (Part B)  Clock Drawing Test  Rey Complex Figure Task – Organisational Score | 🗸  ✓  ✓  ✗  ✓  ✓  ✗  ✗  ✓  ✓  ✓  ✓  ✓  ✓  ✓  ✓  ✓  ✓ |
| Clinical scales  *Stroke severity*  *Degree of disability* | National Institutes of Health Stroke Scale#  Modified Rankin Scale | ✗  ✓ |
| Demographics/mood/quality of life  *Demographic and medical history*  *Recurrent stroke*  *Depression*  *Anxiety*  *Subjective memory*  *Sleep quality*  *Fatigue*  *Quality of life*  *Music experience* | Trial specific demographic and medical history questionnaire  Yes/no  Patient Health Questionnaire-9  Generalised Anxiety Disorder-7 scale  Memory Assessment Clinic Questionnaire  Pittsburgh Sleep Quality Index  Fatigue Assessment Scale  Assessment of Quality-of-Life Scale  Music Experience Questionnaire | ✓  ✓  ✓  ✓  ✓  ✓  ✓  ✓  ✓ |
| Physical fitness  *Cardiorespiratory fitness*  *Sensation and motor ability*  *Subjective daily activity*  *Objective time spent active* | Graded Exercise Test completed on a NuStep  Fugl-Meyer Assessment of Motor Recovery after Stroke (Upper Limb)  Physical Activity Scale for the Elderly  Actiwatch Spectrum Plus worn 24-hours a day over a 7-day period | ✗  ✗  ✓  ✓ |
| Sleep  *Subjective quality/duration*  *Objective sleep/wake patterns* | 7-day sleep diary  Actiwatch Spectrum Plus worn 24 hours a day over a 7-day period | ✓  ✓ |
| Cardiovascular risk@  *Blood pressure*  *ECG-based heart rate variability* | CardXplore worn over a 24-hour period  CardXplore worn over a 24-hour period | ✓  ✓ |
| Genetic testing  *APOE genotyping*  Blood biomarkers  *Inflammatory biomarkers*  *Neurofilament light chain*  *BDNF*  *HbA1c* | Venous blood draw  Venous blood draw  Venous blood draw  Venous blood draw  Venous blood draw | ✗  ✗  ✗  ✗  ✗ |
| Dietary intake | 3-day food diary recorded with Research Food Diary (Xyris Software) | ✓ |
| Gut microbiome | Take home stool specimen collection kit | ✓ |
| *All cognitive tasks have been designed for serial testing and are subject to minimal practice effects. In the case of the ADAS-Cog, HVLT, and MoCA, alternate forms are used on repeat administrations. In addition, the National Adult Reading Task will be administered at one time point to estimate full scale IQ. Performance on individual cognitive tests will be standardised using established norms, and domain scores will be calculated by averaging the standardised scores from each contributing test. #We note that while the NIHSS can be done remotely, the in-person version is more complete and is what we have used for the protocol. @Fitting was in person at study visit but the device was worn for 24 hours by the participant remotely then mailed back in provided envelope.  Note: 3T MRI=3 Tesla Magnetic Resonance Imaging, WAIS-IV=Wechsler Adult Intelligence Scale-4^th^ Edition, ECG= electrocardiogram, APOE= Apolipoprotein E, Inflammatory biomarkers include IL-6, IL-1β, TNF-α, IL-8, IL-10, and IL-1ra, BDNF=Brain Derived Neurotrophic Factor, HbA1c= haemoglobin A1c, ✗=remote assessment not available; ✓=remote assessment available. | | |
