# Supplementary material for: ZOom Delivered Intervention Against Cognitive decline (ZODIAC) COVID-19 pandemic adaptations to the Post-Ischaemic Stroke Cardiovascular Exercise Study (PISCES): protocol for a randomised controlled trial of remotely delivered fitness training for brain health
Source: Trials. 2024 May 18;25:329. doi: 10.1186/s13063-024-08154-1 (PMC11102145; doi:10.1186/s13063-024-08154-1)
Supplement: Supplementary file 2 — Supplementary Material 2. [file 13063_2024_8154_MOESM2_ESM.docx]

**Supplementary Table 2.** An example week of exercise for the intervention and active-control groups.

|  | **Monday** | **Tuesday** | **Wednesday** | **Thursday** | **Friday** |
| --- | --- | --- | --- | --- | --- |
| **Cardiorespiratory intervention** | *Interval and strength session*  Intervals, 4-8 reps: 1 minute on 3 minutes off. Strength exercises, 3x8 reps:  Sit-to-stands, push ups, triceps extension, bicep curls | **No Session** | *Continuous session*  10-30 minutes cycling at a steady state | **No Session** | *Interval and strength session*  Intervals, 4-8 reps: 1 minute on 3 minutes off. Strength exercises, 3x8 reps:  Calf raises, shoulder press, goblet squat, dead bugs |
| **Balance and stretching** | Balance exercise, 3x30s each:  Heel-toe walking, single leg stand, step taps, agility, pick-up, reach-up  Stretches, 2x30s each side per muscle group: hamstrings, back, shoulders, triceps, latissimus dorsi, calves, glutes, quadriceps | **No Session** | Balance exercise, 3x30s each:  Heel-toe walking, single leg stand, step taps, agility, pick-up, reach-up  Stretches, 2x30s each side per muscle group: hamstrings, back, shoulders, triceps, latissimus dorsi, calves, glutes, quadriceps | **No Session** | Balance exercise, 3x30s each:  Heel-toe walking, single leg stand, step taps, agility, pick-up, reach-up  Stretches, 2x30s each side per muscle group: hamstrings, back, shoulders, triceps, latissimus dorsi, calves, glutes, quadriceps |
| Note: Balance and Stretching exercises performed at <40% Heart rate reserve (HRR), Strength performed at BORG-RPE rating 12 – 15, Intervals performed at 50 – 90% HRR, Continuous session performed at 25 – 60% HRR. HRR is calculated from Heart Rate Peak recorded during sub-maximal exercise testing at baseline. | | | | | |
